# *Anopheles arabiensis* continues to be the primary vector of *Plasmodium falciparum* after decades of malaria control in southwestern Ethiopia

**DOI:** 10.1186/s12936-024-04840-2

**Published:** 2024-01-09

**Authors:** Nigatu Eligo, Teklu Wegayehu, Myrthe Pareyn, Girum Tamiru, Bernt Lindtjørn, Fekadu Massebo

**Affiliations:** 1 Department of Biology, Arba Minch University, Arba Minch, Ethiopia; 2 Clinical Sciences Department, Institute of Tropical Medicine, Antwerp, Belgium; 3 Centre for International Health, University of Bergen, Bergen, Norway

**Keywords:** *Anopheles arabiensis*, *Anopheles sergentii*, *Anopheles* species distribution, Circum-sporozoite proteins, Ethiopia

## Abstract

**Background:**

Investigating the species distribution and their role in malaria transmission is important as it varies from place to place and is highly needed to design interventions appropriate to the site. The current study aimed to investigate the *Anopheles* mosquito species distribution and their infection rate in southwestern Ethiopia.

**Methods:**

The study was conducted in 14 malaria-endemic *kebeles* (the smallest administrative unit), which were situated in eight different malaria-endemic districts and four zones in southwestern Ethiopia. Ten per cent of households in each village were visited to collect adult mosquitoes using Centers for Disease Control and Prevention (CDC) light traps. The larval and pupal collection was done from breeding sites within the villages, and reared to adults. Female mosquitoes were morphologically identified. The head and thorax of adult *Anopheles* mosquitoes were tested for circumsporozoite proteins (CSPs) using ELISA. At the same time, legs, wings, and abdomen were used to identify sibling species using PCR targeting the rDNA intergenic spacers region for species typing of the *Anopheles funestus* group and the internal transcribed spacer 2 region genes for *Anopheles gambiae* complex.

**Results:**

A total of 1445 *Anopheles* mosquitoes comprising eight species were collected. Of 813 *An. gambiae* complex tested by PCR, 785 (97%) were *Anopheles arabiensis*, and the remaining 28 (3%) were not amplified. There were 133 *An. funestus* group captured and tested to identify the species, of which 117 (88%) were positive for *Anopheles parensis*, and 15 (11%) were not amplified. A single specimen (1%) showed a band with a different base pair length from the known *An. funestus* group species. Sequencing revealed this was *Anopheles sergentii*. Among 1399 *Anopheles* tested for CSPs by ELISA, 5 (0.4%) *An. arabiensis* were positive for *Plasmodium falciparum* and a single (0.07%) was positive for *Plasmodium vivax*.

**Conclusions:**

*Anopheles arabiensis* continues to play the principal role in malaria transmission despite implementing indoor-based interventions for decades. Sequencing results suggest that *An. sergentii* was amplified by the *An. funestus* group primer, producing PCR amplicon size of different length. Therefore, relying solely on amplifying a specific gene of interest in grouping species could be misleading, as different species may share the same gene.

## Background

Female *Anopheles* mosquitoes can carry *Plasmodium* protozoan parasites, some of which transmit to humans. The majority of malaria caused by *Plasmodium falciparum* and *Plasmodium vivax* occurs in Africa. Between 2010 and 2017, there was a noticeable decline in malaria deaths and cases [1]. However, the decline has slowed in recent years, and in the most recent years, there has been an increase in the number of cases [2]. The presence of the most effective vectors, such as *Anopheles gambiae*, *Anopheles arabiensis*, and *Anopheles funestus* is the main reason for the high rate of malaria transmission in Africa [3]. Other locally important malaria vectors include *Anopheles melas, Anopheles merus, Anopheles moucheti*, and *Anopheles nili* [3].

In Ethiopia, *P. falciparum* and *P. vivax* are the predominant malaria parasites. The country contributed 9% of the *P. vivax* malaria cases globally in 2017 [1]. Although there are many *Anopheles* species in Ethiopia, only a few are known to transmit malaria. *Anopheles arabiensis*, one of the *An. gambiae* complexes are primarily responsible for transmitting malaria in the country [4, 5]. In the 1930s, Italian malariologists identified the “*An. gambiae*” complex as the main vector of malaria in Ethiopia [6, 7]. Ethiopian Malaria Eradication Service, in collaboration with Jolivet, identified 31 *Anopheles* species in 1963 and documented the predominance of “*An. gambiae*” (probably *An. arabiensis*) [7]. *Anopheles gambiae* remained the most common and prominent malaria vector even after extensive malaria vector control interventions were adopted in the 1960 and 1970s [7, 8]. Several recent studies have demonstrated similar contributions to malaria transmission and the dominance of the same species despite decades of malaria control efforts [4, 8–11].

*Anopheles arabiensis* shows flexible resting and feeding behaviour, as it rests and bites indoors and outdoors and feeds on humans and animals based on the hosts’ availability [12]. This behavioural plasticity complicates malaria control and elimination programmes [13]. Despite the plasticity of feeding and resting behaviour, insecticide-treated nets (ITN) and indoor residue spraying (IRS) have been widely used to control this species [14]. *Anopheles* mosquito species diversity and behaviour will likely alter due to these interventions [15–17]. For instance, a shift towards outdoor biting and resting vectors could result from interventions that target indoor feeding and resting vectors [13, 15]. Behavioural resistance, such as avoiding insecticides on bed nets and walls, may reduce the efficacy of IRS and ITNs [16]. Several reports have documented the outdoor and early-night human biting behaviour of *An. arabiensis* in Ethiopia [10, 17]. Integrated interventions may be necessary to target changes in vector species and their behaviour.

Several studies have been conducted to describe the species composition, feeding and resting habits, and infection rates of malaria mosquitos in various malaria-endemic areas [4, 10, 11]. Nevertheless, it has yet to be explored if these malaria control interventions affected the diversity of malaria vector species, their geographic distribution, or infection rates. Therefore, studying the species distribution and their role in malaria transmission is critical for making evidence-based decisions. The current study assessed the composition of the *Anopheles* mosquito species and their relative contribution to malaria transmission in southern Ethiopia.

## Methods

### Study area description

**Fig. 1:**
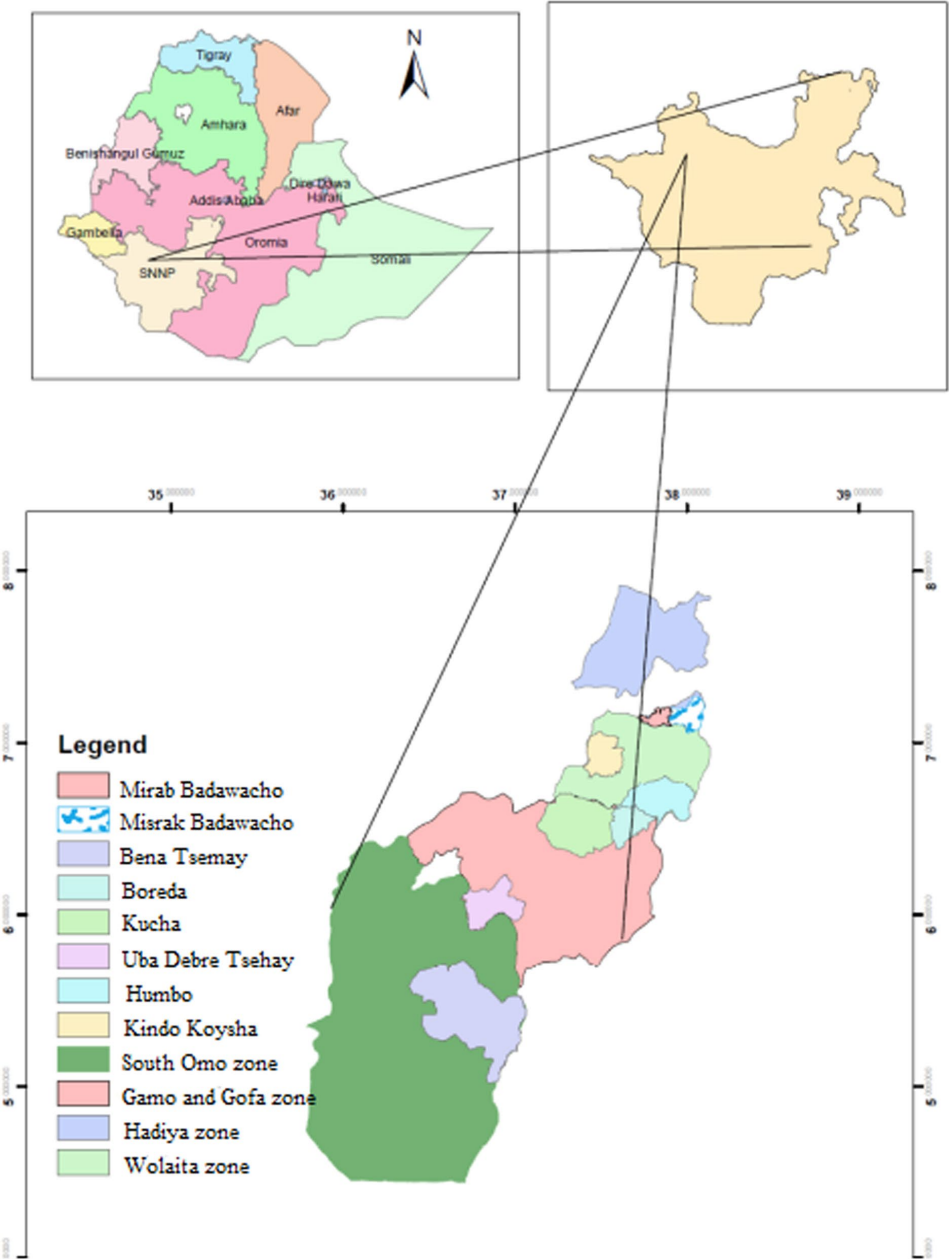
Map of the study zones and districts in Southwest Ethiopia

The study was conducted in four zones of Southern Nation Nationalities and Peoples Regional States (SNNPRs), southwestern Ethiopia (Fig. 1), namely Gamo-Gofa, Wolaita, South Omo and Hadiya. Hot and semi-arid areas comprise 57% of the SNNPRs, tropical sub-humid areas contribute 34%, and tropical humid areas comprise 9%. The region has an elevation of 376 to 4207 m above sea level. Annual rainfall and temperatures range between 500 and 2200 mm and 15 °C and 30 °C, respectively. Despite malaria being a health problem in the region, the incidence of malaria varies from place to place [18].

### Study design and sampling technique

Multistage sampling was used to select districts, *kebeles*, and households. According to the zonal health department report, two high-malaria-prevalence districts in each zone were included, except the Gamo-Gofa zone, where three districts were included. Gamo-Gofa zones were initially together but became two independent zones during the study period. Of the three districts included in the Gamo-Gofa zone, two were located in the present Gamo zone and one was located within the present Gofa zone. Finally, two malarious *kebeles* were purposefully selected in each district based on the malaria incidence. During the study, one district in the South Omo zone was excluded because of a security concern.

Using a random sampling technique, 10% of the households in each *kebele* were selected for adult mosquito collection and larval surveillance close to the households. Systematic sampling was carried out using the lists of households in each *kebele* health post. The first household was selected from one end of the *kebele*, and every tenth household was included. Few houses were replaced by nearby houses when the selected household heads were absent or did not volunteer to participate in the study.

### Sampling adults and larvae of *Anopheles* mosquitoes

Host-seeking *Anopheles* mosquitoes were collected indoors using CDC light traps instead of human landing catches. This was done to reduce bias due to collector skills and limit the risk of infection for collectors. Traps were installed about 1.5 m above the floor near the foot end of the bed where a person slept under an untreated bed net. The traps were set at 6:00 pm and collected at 6:00 am the following morning [19]. Except for the sibling species, collected female *Anopheles* were morphologically identified to the species level using a taxonomic key [20] and preserved in silica gel at room temperature.

A 300 ml standard dipper was used to collect larvae and pupae once during the study period. Pupae and larvae were moved to a temporary insectary set-up at each location. The immature *Anopheles* mosquitoes were collected and kept in a tray until they developed into pupae. The pupae were then transferred to cages until they emerged as adults. All reared female *Anopheles* mosquitoes were killed by chloroform, morphologically identified [20], and stored with silica gel at room temperature until further processing.

### Molecular identification of sibling species

The species of *An. gambiae* complex and *An. funestus* group were identified through PCR at Arba Minch University’s Medical Entomology laboratory. According to the manufacturer’s instructions, the legs, abdomen, and wings were used for DNA extraction using the NucleoSpin Tissue kit (Macherey Nagel, Germany). For *An. gambiae* complex, the rDNA intergenic spacers (IGS) were targeted as described by Scott et al. [21], while for *An. funestus* group, the internal transcribed spacer 2 (ITS-2) region on the rDNA was targeted using PCR as described by Koekemoer et al. [22].

The PCRs were run on the Biometra T professional gradient Thermocycler (Biometra, the Netherlands). The PCR products were visualised on a 2% agarose gel (Sisco Research Laboratories, India) using a 100 bp ladder (Promega, The Netherlands), and species identification was done based on the PCR amplicon height described by Scott et al. [21] and Koekemoer et al. [22].

Amplicon sequencing of *cytochrome c oxidase I* (*COI*) was performed for the specimen that displayed varying heights in PCR amplicons. The initial amplification was performed for the ITS-2 gene, followed by *COI* amplification for further confirmation. The *COI* sequences were aligned, a consensus sequence was generated, and primers were trimmed off using Geneious software [23]. These were used to compare with reference strains that were uploaded in GenBank using BLAST.

### Circum-sporozoite protein test

The head and thorax of female adult *Anopheles* mosquito were used to test for CSPs of *P. falciparum*, *P. vivax*- 210 and *P. vivax*-247 using ELISA, as described by Beier et al. [24]. The sample was prepared by grinding buffer PBS + IGPAL (Fisher Bioreagents, USA and SIGMA-ALDRICH, USA). Fifty microlitres (50 µl) of 0.20 µg/50 µl PBS Pf and 0.10 µg/50 µl PBS (Pv210 and Pv247 each) monoclonal antibodies (mAb) (KPL, USA) were coated on a plate and incubated for half an hour. Confirmation of positive results was obtained by retesting the samples. The remaining steps were carried out as specified in Beier et al. [24].

### Data analysis

The Quantum Geographical Information System (qGIS, www.qgis.org openAFRICA) was used to develop the map. The circumsporozoite protein rate was calculated as the proportion of mosquitoes that tested positive for CSPs in relation to the total number screened. To create a phylogenetic tree, mega molecular evolutionary analysis software [23, 25] was used. Consensus sequences were generated for each specimen and then compared to GenBank reference sequences using BLAST. For multiple sequence alignment, we used the Clustal W tool and trimmed the primers to ensure that all sequences had equal length [23, 25]. Pairwise comparisons were made using *COI* sequences retrieved from GenBank. The inter-species distances were accurately calculated.

## Results

### *Anopheles* species composition

A total of 1445 *Anopheles* mosquitoes were collected. Based on the morphological identification, seven species were documented in the region. Overall, 1227 (85.0%) mosquitoes belonged to the *An. gambiae* complex (46 reared from larvae and 1181 adults collected) and 133 (9.2%) were *An. funestus* group. The remaining *Anopheles* mosquitoes were *Anopheles pharoensis*, *Anopheles pretoriensis*, *Anopheles demeilloni, Anopheles kingi* and *Anopheles tenebrosus* (Table 1). From the larval collection, only 46 *An. gambiae* complex were identified in three study villages.

**Table 1:** Number and proportion of morphologically identified *Anopheles* mosquitoes collected by CDC light traps and larval sampling in southwest Ethiopia

Species	Adult		Immature		Total	
	N	%	N	%	N	%
*Anopheles* mosquitoes						
*An. gambiae* complex	1181	84.4	46	100	1227	84.9
*An. funestus* group	133	9.5	0	0	133	9.2
*An. pharoensis*	70	5.0	0	0	70	4.8
*An. pretoriensis*	9	0.6	0	0	9	0.6
*An. demeilloni*	3	0.2	0	0	3	0.2
*An. kingi*	2	0.1	0	0	2	0.14
*An. tenebrosus*	1	0.07	0	0	1	0.07
Total	1399		46		1445	100

### Spatial distribution of *Anopheles* mosquitoes

*Anopheles gambiae* complex was the most common and widely distributed species in the region, occurring in each village except in Mirab Badawacho of Hadiya zone (Table 2). *Anopheles pharoensis* was observed in 50% of study sites. *Anopheles funestus* group was collected only from Humabo of Wolaita zone. *Anopheles demeilloni*, *An. kingi*, and *An. tenebrosus* were rare species.

**Table 2:** Number of *Anopheles* mosquitoes collected by CDC light traps and larval sampling in different districts southwest Ethiopia

Variables	Gamo zone		Gofa zone	Wolaita zone		South Omo zone	Hadiya zone		Total
	Kucha (1292)	Boreda (1692)	Uba D/Tse (1087)	Humbo (1212)	K/K (1219)	Bena/T (555)	Misrak/B (1733)	Mirab/B (1763)	
*Anopheles* species									
*An. gambiae complex*	47	8	48	34	125	39	926	0	1227
*An. funestus* group	0	0	0	133	0	0	0	0	133
*An. pharoensis*	0	0	1	62	0	0	5	2	70
*An. pretoriensis*	4	2	1	0	2	0	0	0	9
*An. demeilloni*	0	1	0	2	0	0	0	0	3
*An. kingi*	0	0	1	0	1	0	0	0	2
*An. tenebrosus*	0	0	0	0	0	1	0	0	1
Total	51	11	51	231	128	40	931	2	1445

Uba D/Tse = Uba Debretsehaye; K/K = Kindo Koyisha; Bena/T = Bena Tsemay; Misrak/B = Misrak Badawacho; Mirab/B = Mirab Badawacho; The numbers in parentheses represent altitude in metres above sea level

### Sibling species of *An. gambiae* complex and *An. funestus* group

Out of the 767 *An. gambiae* sensu lato (s.l.) adults tested, 97% (743) were successfully amplified for *An. arabiensis*, and of the 46 *An. gambiae* s.l. specimens that emerged from larvae, 91% (42) were amplified for *An. arabiensis*. The remaining specimens (4 from larval collection and 24 from adult collection) were not amplified.

**Fig. 2:**
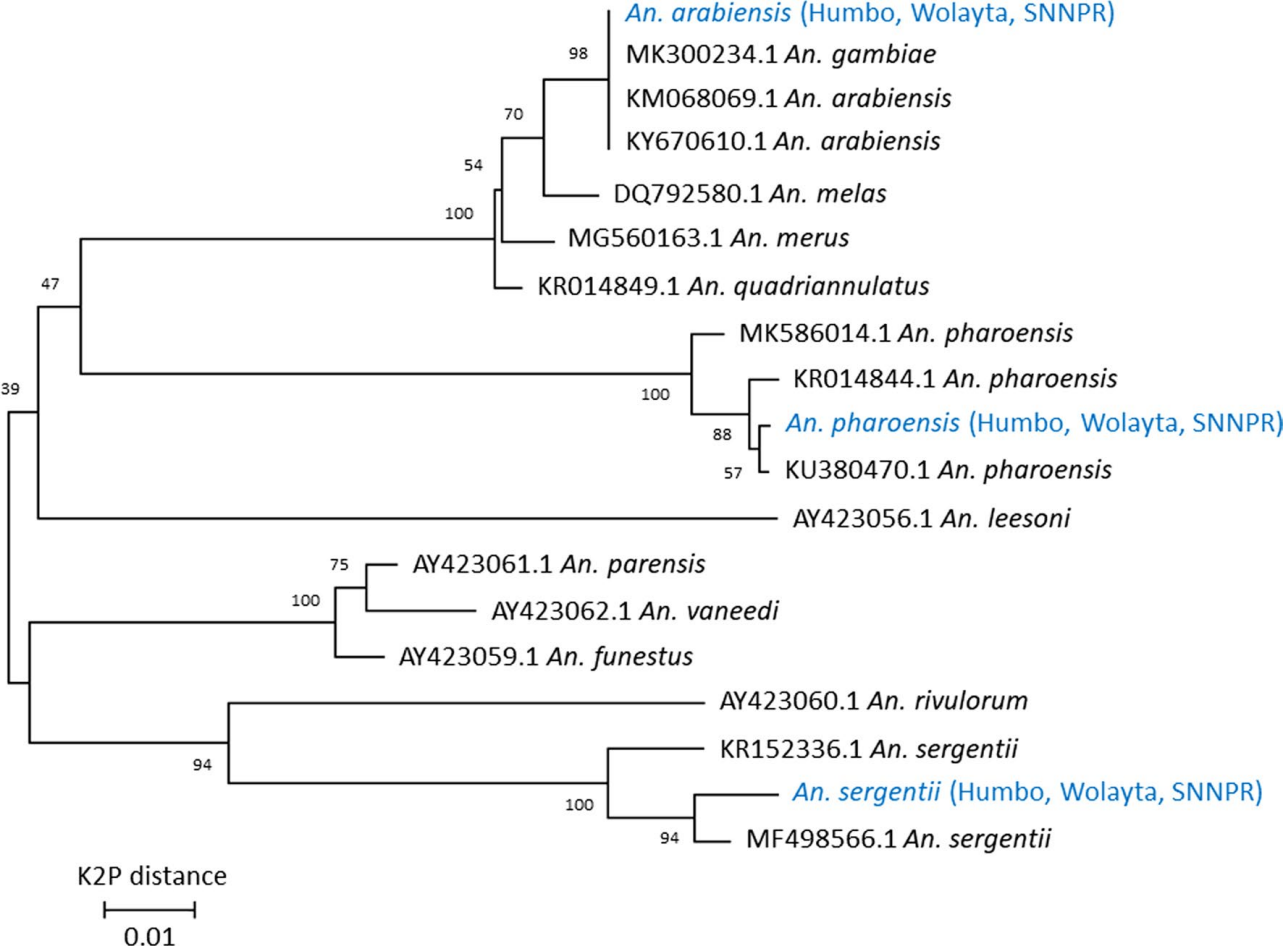
Phylogenetic tree of *Anopheles funestus* group and two other species collected the study sites. The variance of the strain *An. sergentii* species identified from the Humbo district of the Wolaita zone of southwest Ethiopia and other *An. sergentii* strains KT160247 from Egypt: Fayoum, and KT160246 from Egypt: Aswan. Based on a pairwise comparison of the COI sequence of *An. funestus* and *An. sergentii* retrieved from GenBank, it can be inferred that the current species belong to the *An. sergentii* group, rather than the *An. funestus* group. The comparison revealed that the percentage relation between the COI sequence of *An. funestus* and the species of interest was 82%, while for *An. sergentii*, it was more than 95%

Of the 133 *An. funestus* s.l. tested, 87.9% (117/133) were amplified as *An. parensis*, and 15 (11%) were not amplified. A single specimen (0.7%) collected in the Humbo district of the Wolaita zone was amplified using primers for the *An. funestus* group, but its PCR amplicon height was not in accordance with the existing group members. The standard PCR amplicon size for *An. funestus* s.l. was 505 bp for *An. funestus* sensu stricto (s.s.), 587 bp for *An. vaneedeni*, 411 pb for *Anopheles rivulorum*, 313 bp for *An. rivulorum*-like, 252 bp for *An. parensis*, and 146 bp for *Anopheles leesoni*. The species of interest had a PCR amplicon size of 200 bp. After sequencing, the specimen was identified as *Anopheles sergentii* (Fig. 2).

### *Plasmodium* sporozoite infection rate of *Anopheles* species

Of the 1399 *Anopheles* mosquitoes tested for *Plasmodium* CSPs, six *An. arabiensis* were positive for CSPs with an overall prevalence of 0.43% (95% Confidence Interval (CI): 0.16–0.93). Of the six positive *An. arabiensis*, five were positive for *P. falciparum* CSP (0.36%; 95% CI 0.11–0.83), and 1 was positive for *P. vivax* (0.07%; 95% CI 0.002–0.40)—none of the other mosquito species tested positive for CSPs.

## Discussion

Eight *Anopheles* species, namely *An. arabiensis*, *An. parensis*, *An. pharoensis*, *An. pretoriensis*, *An. demeilloni*, *An. kingi*, *An. tenebrosus* and *An. sergentii*, were identified in southwest Ethiopia. *Anopheles arabiensis* was the most common and primary malaria vector in the region. There were no documented cases of *Anopheles stephensi* in the region.

*Anopheles arabiensis* was the region’s most widely distributed and predominantly vectoring *P. falciparum* malaria. Many years ago, O’Connor [26] documented 34 *Anopheles* species in Ethiopia. Among these *Anopheles* species recorded at that time, *An. gambiae* (presumably *An. arabiensis*) was the dominant and primary malaria vector in wide geographic areas. Several studies have also shown that *An. arabiensis* contribution as a primary vector in different parts of the country [5, 10, 11]. Despite implementing a wide range of vector control interventions for decades, the species remained the dominant malaria vector in the region. For example, the *P. falciparum* CSP infection rate of *An. arabiensis* was 0.36%, comparable to the CSP rate of 0.77% reported from Gambella in 1994 [27]. Krafsur reported a somewhat higher CSP rate (1.87%) of the same species from Gambella in 1977, although the method was a microscopic dissection of salivary glands [6]. Using the HLC technique, the *P. falciparum* CSP rate of *An. arabiensis* was 0.5% in Sille, southern Ethiopia [28]. In the Central Rift Valley of Ethiopia, a CSP rate of *An. arabiensis* was 1.18% from the CDC light traps [29]. The *P. falciparum* CSP rate of *An. arabiensis* from CDC light traps, as used in the current investigation, was 0.3% in the south-central and southwest Ethiopia [4, 30, 31]. This implies that *An. arabiensis* does not respond well to the current malaria vector control interventions. To effectively eliminate malaria in the country, it is necessary to use additional tools to control the species [32, 33]. For example, interventions such as larval source management can be used to target the aquatic stages of malaria and other vectors [34]. Toxic bait traps could be used to control mosquitoes seeking hosts outdoor [32], while animal-based interventions can address the problem related to zoophagic mosquitoes [35].

A challenge with *An. arabiensis* is its plasticity in feeding and resting habits. For example, the species’ anthropophagic, zoophagic, and outdoor and indoor resting behaviour has been documented in Ethiopia [12, 36, 37]. These adaptive characteristics allow the species to avoid interventions mainly targeting those that rest and feed indoors.

The majority of *An. funestus* group was identified as *An. parensis*. Similarly, *An. funestus* group from Jimma was analysed and verified as *An. parensis* using the PCR technique [38], suggesting that *An. parensis* is the predominant species of *An. funestus* group in the region. During the 1930 and 1960s, the *An. funestus* group was prevalent and widespread in Ethiopia [39]. However, none of the 339 *An. funestus* group mosquitoes from Zwai and Awasa tested positive for sporozoite [39]. In contrast, Krafsur reported a sporozoite rate of 1.23% in Gambella using PSCs in 1977 [8]. However, identifying the specific species within the group was challenging due to the limited identification tools available. It’s worth noting that *An. funestus s.s.* tends to bite and rest indoors, making it a potential candidate for DDT IRS, as seen in East African countries [39]. Following the widespread use of DDT IRS, the outdoor biting and resting species *An. rivulorum* substituted the primary vector *An. funestus s.s.* in East Africa [40]. This species was not identified at the current study sites. During the 1960s, the IRS projects carried out in southern Africa resulted in the elimination of the same species [41]. This suggests that indoor-based intervention methods could be effective in reducing malaria vectors if the vectors are completely resting and biting indoors. However, in the presence of opportunistic feeders like *An. arabiensis*, the current interventions may need to be revised to achieve the intended objective of malaria control and elimination.

In this study, after a detailed analysis of the sequence, a single *Anopheles* mosquito specimen was confirmed to be *An. sergentii*. Two species of *An. sergentii* have been documented in Africa: *An. sergentii sergentii*, found in several countries, and *An. sergentii macmahoni*, mainly found in East Africa, including Ethiopia [42]. Adult *An. sergentii macmahoni* are rarely found indoors in human dwellings and typically feed on animals [3]. *Anopheles sergentii sergentii* is an important malaria vector in the Saharan belt, spanning from northern Africa to the Middle East [3]. The species is morphologically related to *An. parensis*, *An. funestus*, *An. demeilloni* and *An. cameroni* [43]. It was also grouped under *An. demeilloni* in the report of “Phylogeny and Classification of *Anopheles*” [44]. It is important to note that although the primers of the *An. funestus* group amplify *An. sergentii*, it is insufficient evidence to classify it as a member of the *An. funestus* group. However, the results from sequencing suggest that the two species share the gene of interest used to identify species in the *An. funestus* group. Therefore, it is crucial to provide detailed information on the degree of species divergence and percentage relationship by comparing the sequence of the species of interest with the sequence of the species obtained from GenBank [45] in addition to primer amplification. By comparing *COI* sequences from GenBank, it is evident that the current species belongs to *An. sergentii* and does not belong to *An. funestus* group.

The cross-sectional method used in this study has limitations because it cannot account for monthly and seasonal variations in species composition, which could lead to an incomplete list of species. Additionally, no confirmatory molecular test or boiling was performed on CSP ELISA positive samples, which could have led to false positives. However, the positive samples were repeated using the ELISA assay.

## Conclusions

Despite several decades of efforts to deploy indoor-based interventions, *An. arabiensis* continues to play a dominant role in transmission. Based on the sequencing data, the *Anopheles* species amplified for *An. funestus* group primers were identified as *An. sergentii*. It is important to note that there were no documented cases of *An. stephensi* in the study sites.

## Data Availability

The article contains the data that was utilised to draw the conclusion.
